# The state of cancer research in fragile and conflict-affected settings in the Middle East and North Africa Region: A bibliometric analysis

**DOI:** 10.3389/fonc.2023.1083836

**Published:** 2023-03-23

**Authors:** Zahi Abdul Sater, Theresa Farhat, Mohamed N. Elsayed, Yara Youssef, Marium Husain, Malak Kaddoura, Lubna Jaber, Deborah Mukherji, Ali Taher

**Affiliations:** ^1^ Global Health Institute, American University of Beirut, Beirut, Lebanon; ^2^ College of Public Health, Phoenicia University, Mazraat El Daoudiyeh, Lebanon; ^3^ Department of Internal Medicine, James Comprehensive Cancer Center, Ohio State University, Ohio, United States; ^4^ Department of Internal Medicine, American University of Beirut Medical Center, Beirut, Lebanon

**Keywords:** cancer research, bibliometric, conflict settings, Arab world, MENA region

## Abstract

**Background:**

Cancer represents a disproportionate burden in LMICs, especially conflict-affected countries in the MENA region. Research output on cancer fails to match the growing burden in the region. This bibliometric study aims to examine the status and trends of cancer research in fragile and conflict-affected settings in the MENA region from 2000 to 2021, while also incorporating economic and demographic indicators as additional factors of analysis.

**Methods:**

The Web of Science databases were searched for publications related to cancer research in Iraq, Lebanon, Libya, Palestine, Syria, and Yemen from January 1, 2000, to December 31, 2021. The retrieved publications were screened based on preset eligibility criteria and the final list was analyzed using the Bibliometrix Package in R to generate the annual scientific production and citations, journals, institutions, authors, collaborations, keywords, and title co-occurrence. Each country’s annual scientific production was analyzed against its annual GDP per capita.

**Results:**

A total of 4,280 documents met the inclusion criteria in this research. The annual number of publications revealed a significant increase over the past 20 years. These publications were mostly published in international journals that had impact factors rated in the 3rd or 4th quartiles. The overall contribution of researchers from Fragile and Conflict-Affected Settings (FCS) to cancer research was 6.5% of the MENA cancer research productivity, despite comprising around 23% of the total MENA region’s population. Lebanon had the highest publication productivity at the country level, followed by Iraq and Syria. GDP per capita was not significantly correlated with cancer research across the countries under investigation. At the institutional level, the American University of Beirut was the most prolific institution and had the highest number of collaborations and the widest range of cooperative partners. Most first authors were male researchers. There is an interest in cancer expression, prevalence, diagnosis, and management in terms of commonly researched topics.

**Conclusion:**

This study underscores the need for a concerted effort to improve cancer research outcomes in FCS, which can be achieved through targeted research, increased investment in research infrastructure and capacity-building initiatives, and greater regional and global collaboration.

## Introduction

The global burden of cancer is disproportionately higher in low- and middle-income countries (LMICs) compared to high-income countries (1–4). It is speculated that by 2035, two-thirds of the world’s cancer cases will arise in LMICs, which include many countries in the Middle East and North Africa (MENA) region (5). To address this burden, it is important to establish regional and national cancer control plans, strengthen cancer surveillance systems, build clinical and research capacity, and promote cancer research (2). Locally driven and high-quality cancer research is essential to understand the challenges and barriers to the cancer care continuum and address them in effective, sustainable, and context-specific approaches.

The MENA region’s capacity to conduct research is limited compared to other regions. The recurrent and endemic nature of conflicts and political and economic instability in many MENA countries has resulted in numerous systemic challenges to healthcare research, including cancer research. Currently, ongoing conflicts in Iraq, Libya, Palestine, Yemen, and Syria, and the resultant mass displacement, have led to the worst humanitarian crisis since World War II (6). In 2021, 18% of all forcibly displaced people worldwide originated from the MENA region (7). With its fragile health system, Lebanon currently hosts the world’s highest number of refugees per capita following the Syrian crisis (8). Regional conflicts and crises, as well as Lebanon’s civil war, Israel’s Second Lebanon War, and protracted internal struggle have all had an impact on its politics and socioeconomic situation (9).

Although health research in active conflict settings is crucial, it is often deprioritized as the focus shifts to resolving conflict, delivering humanitarian assistance, and managing forced displacement (10). Insufficient spending on research, poor research and health infrastructure, fragmented health systems with underdeveloped disease registries, and limited collaboration with the global research community undermine the capacity to conduct locally relevant health research in these contexts, particularly cancer research (2, 3, 11). In addition, the lack of research culture, which can be attributed to cultural norms in some countries, negatively impacts cancer research despite its importance and relevance to local challenges (12, 13). Our knowledge of the causes of cancer, our ability to control it, and how it is impacted by conflict would advance considerably if the clinical, basic, environmental, lifestyle and ethnic determinants of cancer were thoroughly investigated (14).

Despite the challenges mentioned above, the MENA region has been actively contributing to cancer research over the past two decades (15–18). However, and to the best of our knowledge, studies using bibliometric methods to analyze the knowledge output and trends of research on cancer in Fragile and Conflict-Affected Settings (FCS) in the MENA region are limited. Against this backdrop, we conducted a bibliometric study of published cancer research between 2000 and 2021 in FCS in the MENA region: Iraq, Lebanon, Libya, Palestine, Syria and Yemen. Our study investigated the trend of publications over the study period and the characteristics of published research, including the authors’ institutional affiliations and collaborative landscape. Demographic and economic indicators were also considered as we compared research output across the study countries.

## Methods

### Source of data

Scientific publications focusing on cancer research in Fragile and Conflict-Affected Settings (FCS) in the MENA region, as defined by the World Bank (19) were searched in the Web of Science (WoS) database on June 30, 2022. WoS is the most relevant, prominent, extensive, and reliable database for literature retrieval and analysis (20, 21). Data on the study countries’ sociodemographic and economic indicators were retrieved from the World Bank’s DataBank (22).

### Search strategy

A search strategy (**Supplemental Table 1**) was developed using an extensive list of cancer-related keywords compiled from previous studies, reviews, practical reporting, and meta-analyses to search for titles, and abstracts listed under WoS oncology categories and published in oncology-specific journals. Then, the six FCS (Iraq, Lebanon, Libya, Palestine, Syria, and Yemen) were entered as country affiliations. Four indices were used: Science Citation Index Expanded (SCI-EXPANDED), Social Sciences Citation Index (SSCI), Arts and Humanities Citation Index (A&HCI), and Emerging Sources Citation Index (ESCI).

### Inclusion criteria

Cancer research publications with at least one of the authors from FCS in the MENA region were included. Publications that did not primarily relate to cancer were excluded. The period of the index date was from January 1, 2000, to December 31, 2021, with the language restricted to English. The document types included were original articles, article data, and article early access. All other types of articles including review articles, case reports, book chapters, and letters to the editor were excluded (**Supplemental Figure 1**).

### Data management and selection process

Full metadata of the articles identified was downloaded in text format and then imported into an excel file. Two reviewers independently screened the titles and abstracts of retrieved publications and discrepancies were resolved by discussion with a third reviewer. A full-text review of all included references was also conducted independently by the two reviewers. A second full-text review was performed on the discrepant articles to make the final decision for inclusion/exclusion.

### Data extraction and analysis

The analysis of the final list of publications was completed using the Bibliometrix Package (http://www.bibliometrix.org/), which is an R statistical software package for comprehensive science mapping analysis (23). The raw data exported from R was transformed into graphic and tabular using the Flourish software (24) to generate the annual scientific production and citations, journals, count, affiliations, corresponding authors’ countries, the gender distribution of authors by countries, collaboration patterns, universities’ collaborations, and author’s keywords and title co-occurrence. The gender of the authors was determined using the gender API (gender-api.com), which uses the author’s first name and country of origin as inputs. Authors with no scores or scores <90 and first names shorter than 2 letters were filtered out. The impact factor (IF) and quartiles (Q) of journals were derived from the Journal Citation Reports (JCR; Web of Knowledge) in October 2022 (25).

### Ethical approval

The study did not require the submission of an IRB application since the researchers used publicly available information and did not involve any interactions with human participants.

### Statistical methods

Data from *WoS* were exported to *Excel* and then to *PRISM version 9* for analysis, where relevant. Categorical data were expressed as percentages. Data on the study countries’ growth domestic expenditure on research and development could not be obtained from online public resources. Therefore, we used the countries’ growth domestic product (GDP) in the current US$ as a proxy for their economic positions and analyzed the trend of publications from each country against its annual GDP per capita, as reported in the World Bank’s public databases (26). We performed linear regression analysis to assess the statistical significance of the increasing trend in annual publications between 2000 and 2021. The model included the number of publications each year (as the dependent variable) and the year (as the independent variable). We performed a similar analysis to assess any statistically significant correlation between each country’s GDP per capita and yearly publications over the study period. P<0.05 was considered to indicate statistical significance in both models.

## Results

### Publications analysis

**Figure 1:**
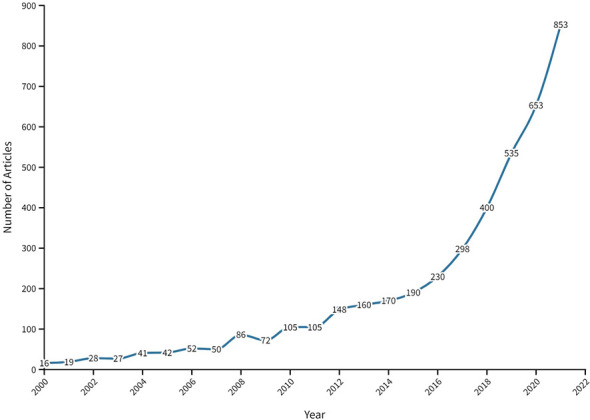
Annual number of cancer research publications in FCS in the MENA region from 2000 to 2021.

**Figure 2:**
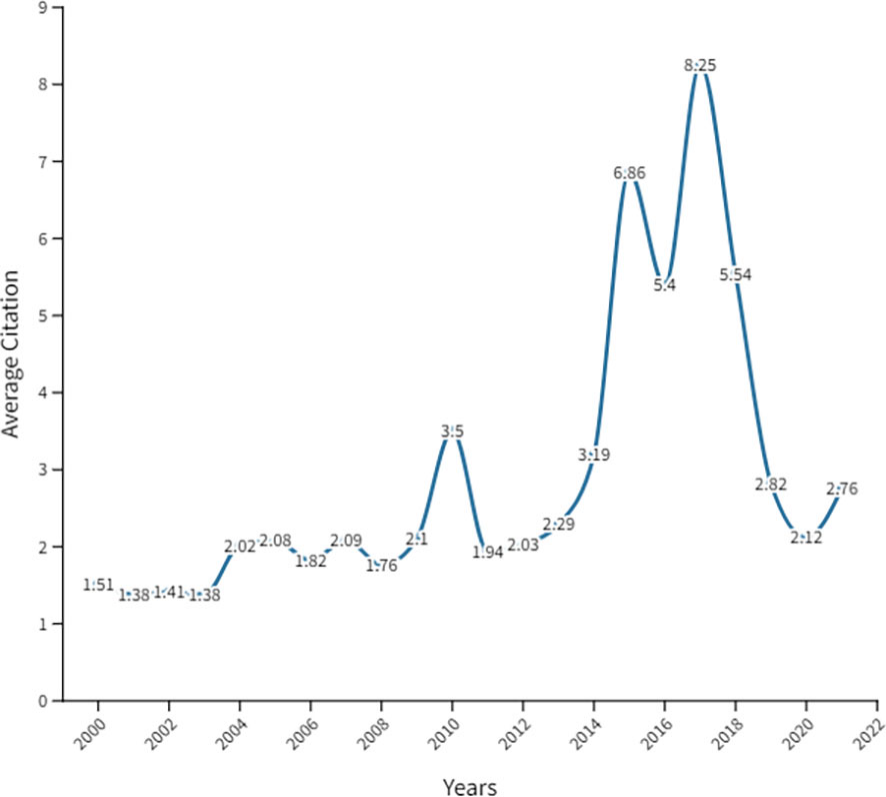
The average annual citations of cancer research publications in FCS in the MENA region from 2000 to 2021.

Our search for cancer research publications in FCS countries in the MENA region between 2000 and 2021 yielded 5,064 publications, out of which 4,280 were included, based on the eligibility criteria (**Supplemental Figure 1**). The annual number of publications significantly increased (P<0.0001) from 16 in 2000 to 853 in 2021 (**Figure 1**). The data indicated an inconsistent rising trend in the number of publications, with an average annual change rate of approximately 22% over 13 years (2000-2013). From 2013 to 2021, the number of publications increased steadily, reaching a peak in 2021 (**Supplemental Figure 2**). The overall contribution of authors from FCS to cancer research productivity in the MENA was 6.5% (data not shown). Notably, the average annual citations per publication significantly increased from 2000 to 2021 (P<0.0002), as presented in **Figure 2**. The yearly average article citation ranged from 1.51 and 3.19 between 2000 and 2014, with a sharp increase to 6.86 citations in 2015 and a peak of to 8.25 citations per publication in 2017, followed by a decline to around 2.12 citations in 2020 (**Figure 2**). On average, the total number of citations per publication was around 18.1 (**Supplemental Table 2**).

### Analysis of sources

**Figure 3:**
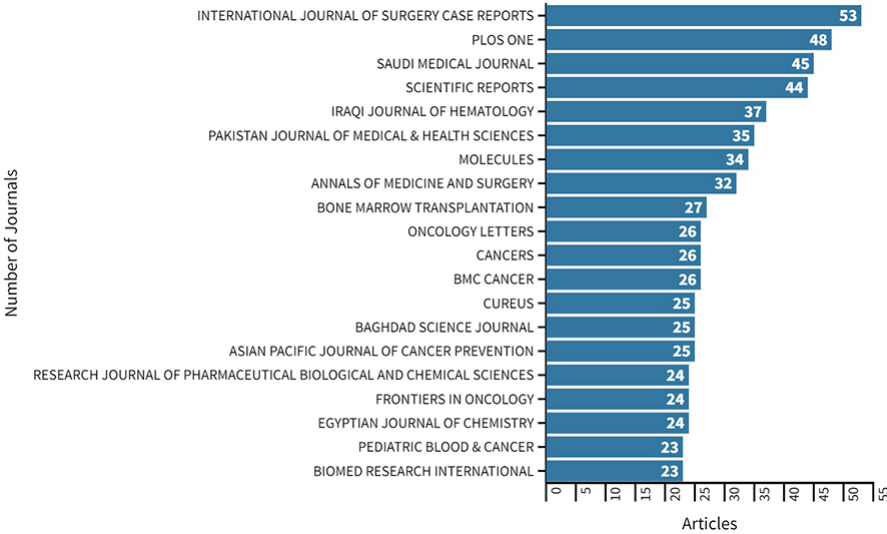
Top 20 most relevant sources by the number of publications on cancer research in FCS in the MENA region from 2000 to 2021.

The retrieved documents were published in 1,514 sources, including journals and books among others. The *International Journal of Surgery Case Reports*, with an impact factor (IF) of 0.690 in 2021, was the most used source for publication of cancer research in FCS (53 publications; 1.23%). This was followed by *PLOS One* (IF 3.752; 48 publications; 1.12%), the *Saudi Medical Journal* (IF 1.422; 45 publications, 1.05%), *Scientific Reports* (IF 4.996; 44 publications; 1.02%), and the *Iraqi Journal of Hematology* (37 publications; 0.86%) (**Figure 3**, **Supplemental Table 3**). In our study, the top 20 sources published 14.6% (n=626) of the research manuscripts, 41.21% of which were Q2 journals. Only one Q1 journal, the journal of *Cancer*, had a few publications (n=26, 4.15%). Among the top 20 sources, four were based in the MENA region, and the journal with the most publications focus on case reports.

### Analysis of countries and institutions

**Figure 4:**
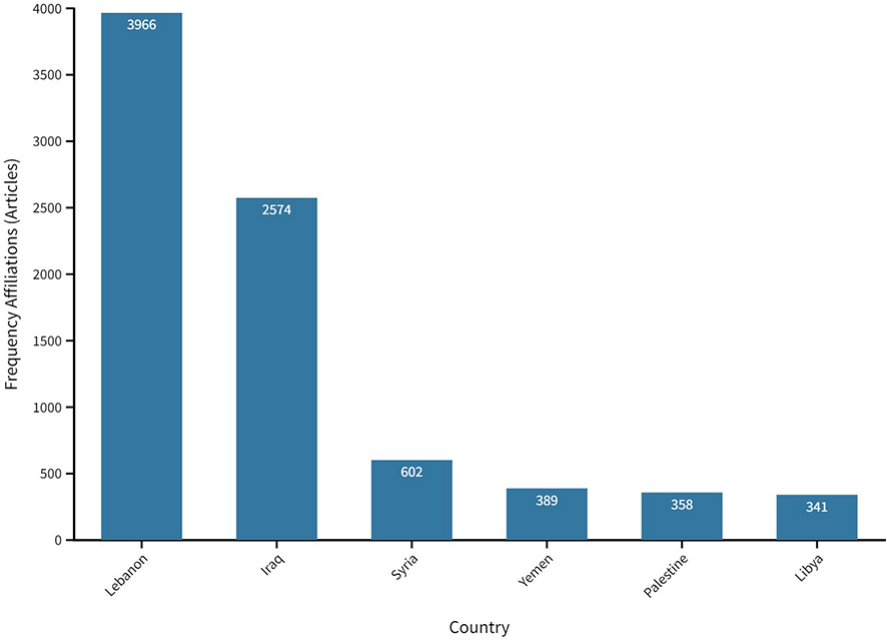
Country production from 2000 to 2021.

Lebanon was the leading country in terms of the number of publications on cancer research, with a total of 3,966 publications and 12,509 citations, and an average article citation of 11.86. Iraq followed with 2,574 (2,888 citations, average article citations of 3.55), then Syria with 602 (825 citations, average article citations 4.10), Yemen 389 (672 citations, average article citations 10.03), Palestine 358, and Libya 341 publications (818 citations, average article citations 12.21) (**Figure 4**).

The GDP per capita in the six countries displayed a high level of fluctuation between 2000 and 2021. Nevertheless, published cancer research steadily increased over the same period. Lebanon had the second-highest median GDP per capita ($6,733) after Libya ($8,590), followed by Syria ($5,157), Iraq ($4,610), Palestine ($2,516), and Yemen ($943) (**Supplemental Figure 3**). There was no significant correlation between the median GDPs per capita and scientific publications on cancer for the six countries collectively or any of the individual countries. As an example, Lebanon, the highest contributor among the study countries, had a correlation coefficient of –0.01 (p value= 0.86) between the number of yearly publications (dependent variable) and annual GDP per capita (independent variable) between 2000 and 2021.

**Figure 5:**
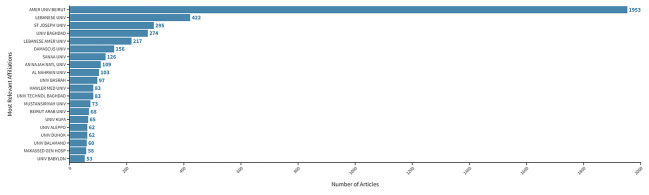
Most relevant affiliations per article on cancer research in FCS in the MENA region from 2000 to 2021. From top to bottom: American University of Beirut, Lebanese University, Saint Joseph University, University of Baghdad, Lebanese American University, Damascus University, Sanaa University, An-Najah National University, Al Nahrain University, University of Basra, Hawler Medical University, University of technology Baghdad, Mustansiriyah University, Beirut Arab University, University of Kufa, University of Aleppo, University of Duhok, University of Balamand, Makassed general hospital, University of Babylon.

The retrieved publications were published by authors from 6,816 different institutions. The top 20 institutions, selected and listed based on the number of publications, included the American University of Beirut (Lebanon; 1,953), followed by the Lebanese University (Lebanon; 422), the Saint Joseph University of Beirut (Lebanon; 295), the University of Baghdad (Iraq; 274), and the Lebanese American University (Lebanon; 217) (**Figure 5**). Four of the top five institutions are from Lebanon.

### Analysis of authors and gender distribution

**Figure 6:**
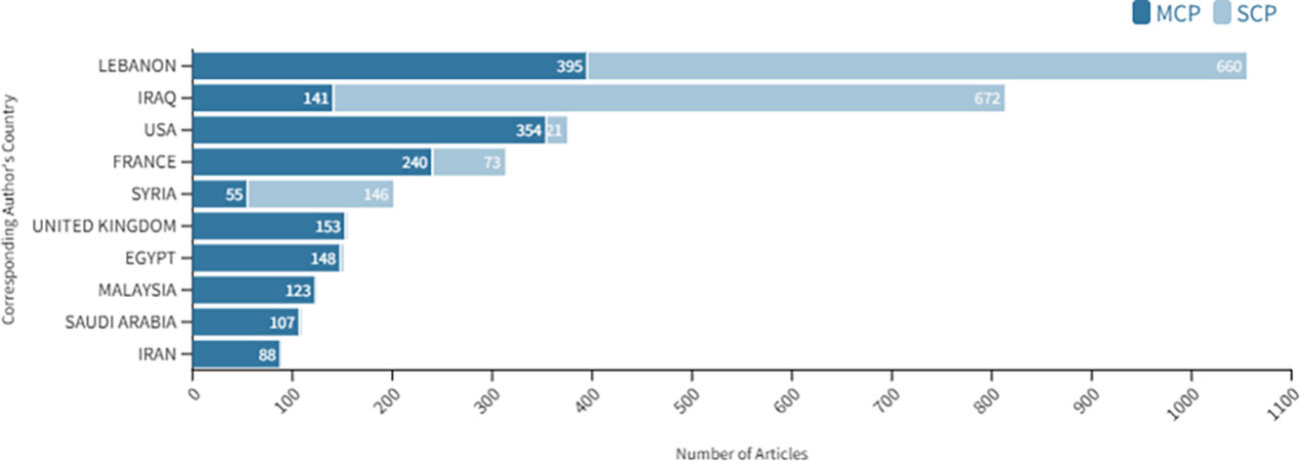
Top corresponding authors**’** countries on cancer research in FCS countries in the MENA region from 2000 to 2021. SCP stands for single-country publication. MCP stands for multiple-country publication.

**Figure 7:**
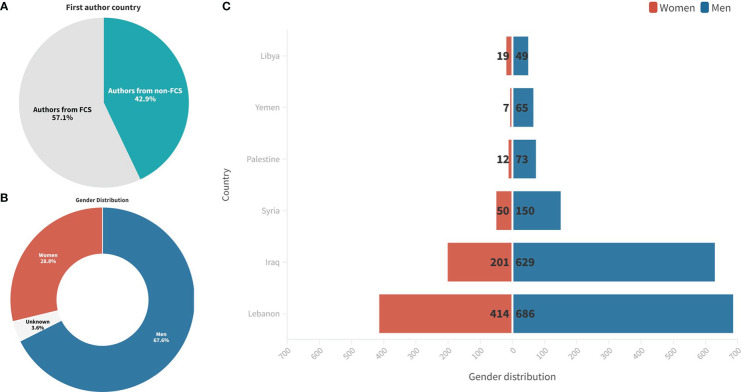
**(A)** Percentage of first authors from FCS compared to non-FCS. Gender distribution of first authors **(B)** and **(C)** by FCS.

From 2000 to 2021, there were a total of 29,246 authors who contributed to the 4,280 publications on cancer research in FCS, with an average of 6.83 authors per document (**Supplementary Table 1**). There were 100 (0.3%) single-authored publications and 29,146 (99.7%) multi-authored publications. The top corresponding authors from FCS who published on cancer research were from Lebanon, Iraq, and Syria. Most of their publications (62.5%, 82.7%, and 72.6%, respectively) were single-country publications (SCP) (**Figure 6**). Around 37% of the corresponding authors were neither from MENA nor FCS (data not shown). A sizeable portion of the first authors (n=1,838 42.9%) of the dataset were from 71 non-FCS countries, mainly the USA, France, England, Saudi Arabia, and Malaysia (**Figure 7A**). Among the first authors from FCS (n=2,442, 57.1%), 67.6% (n=1,652) were men and 28.8% (n=703) were women, while the gender of the rest was unknown (n=87, 3.6%) (**Figure 7B**). The female-to-male gender ratio was below 3 in only Lebanon and Libya (**Figure 7C**).

### Analysis of collaborations

**Figure 8:**
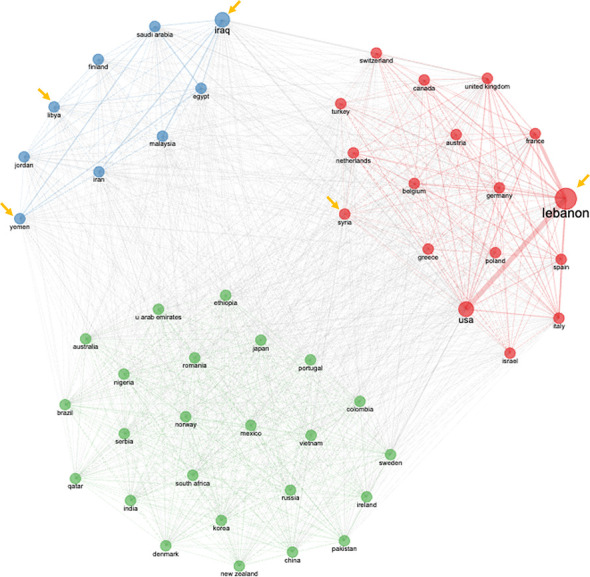
Country collaboration on cancer research in FCS in the MENA region from 2000 to 2021. The dimensions of the rectangles symbolize the number of cooperative partners, while the font size of the countries shows the number of their collaborations. Additionally, the closeness of the countries**’** partnership is reflected by the thickness of the lines. Arrows indicate the FCS countries included in this study.

To further examine the collaborative landscape of cancer research in FCS in the MENA region, an analysis was conducted on the countries’ partnerships. Three main collaboration clusters were recognized in **Figure 8**. The first cluster consisted of Lebanon, the USA, France, Spain, Italy, Canada, Germany, and Syria with Lebanon demonstrating strong collaborations with USA and France. The second cluster included Iraq, Iran, Malaysia, Finland, China, India, Australia, Ethiopia, and the United Kingdom with nonspecific linkages. The third cluster included Saudi Arabia, with its main collaborations being with Egypt and Yemen. These clusters spotlight the cooperative research partnership on cancer in FCS.

### Authors’ keywords and co-occurrences

**Figure 9:**
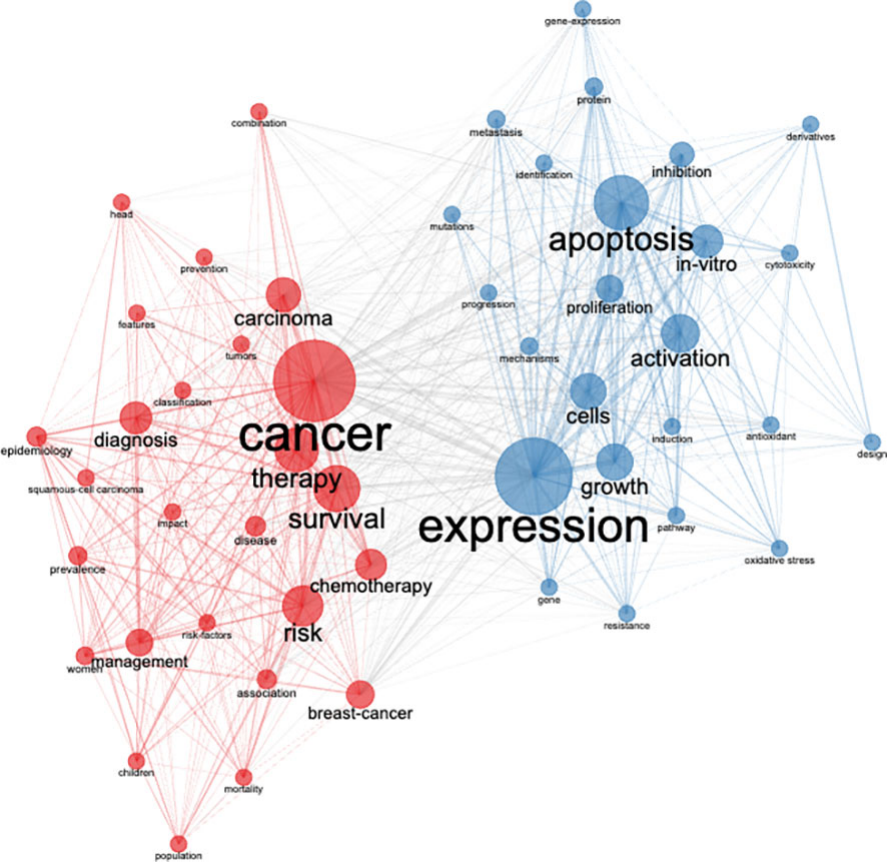
Co-occurrence network of most frequently used author keywords on cancer research in FCS in the MENA region from 2000 to 2021. The rectangle size reflects the total number of highly cited articles, while the color and thickness of the lines reflect the link clustering and strength, respectively.

In general, keywords convey the major idea and subject matter of an article, while their co-occurrence indicates their degree of association and relevance. This study identified around 9,560 keywords, with 162 high-frequency keywords, occurring ≥10 times and forming two clusters, namely “cancer” and “expression” (**Figure 9**). The top-ranked keywords were “breast cancer”, “cancer”, “apoptosis” and “cytotoxicity” (**Supplementary Figure 4**) occurring 329, 183, 156, and 105 times, respectively (data not shown). The first cluster, consisting of interconnected keywords related to “expression”, focused on basic research, and included co-occurring keywords such as apoptosis, growth, cells, activation, proliferation, mechanisms, and others. The second cluster contained interconnected keywords related to “cancer” and “apoptosis” and included keywords related to prevalence, diagnosis, and management such as carcinoma, therapy, chemotherapy, risk, diagnosis, epidemiology, management, and others.

## Discussion

Armed conflicts and insurgencies have severely undermined the research capabilities of several MENA countries. Ranging from chronic underfinancing and poor infrastructure to deficient research culture (15, 18, 27), the challenges health researchers face in fragile and conflict-affected settings are numerous (11, 28). Aiming to identify opportunities for improvement, our study aimed to map out published cancer research and highlight the characteristics of published research across 6 countries affected by conflict in the MENA region.

The study found that there has been a consistent rise in the total number of cancer publications in the six FCS investigated from 2000 to 2021. This trend may reflect the growing concern about cancer as a major public health problem. Nevertheless, this increase is relatively small compared to other countries in the region and worldwide. Additionally, the annual citation rate and regional contribution are relatively low, suggesting a diminished capacity to produce quality research. This could be because researchers lack motivation or support from their institutions in most of these countries. Most publications focused on cancer expression, prevalence, diagnosis, and management. Therefore, targeted research on these topics could help to fill gaps in knowledge and provide insights that can be used to inform cancer control strategies and improve patient outcomes in FCS. Importantly, despite being home to 23% of the total MENA region’s population and constituting 10% of the region’s GDP in current US dollars in 2020 (the latest year reported in the World Bank’s database), the six countries represented only around 6.5% of the MENA region’s research productivity. The studies that were conducted were often collaborative, with the majority involving non-MENA countries as indicated by the countries of the first and last authors. Furthermore, the shortage of funding for authors and the high publication costs could negatively impact research productivity, particularly in conflict-affected settings where resources are already limited. Overall, this highlights the urgent need for greater investment in research infrastructure and capacity-building initiatives, and for funding agencies to provide more support for cancer research in FCS.

The need for more robust study designs that can provide higher quality evidence is highlighted by the high number of case reports published in the *International Journal of Surgery Case Reports*, a journal focused on case reports. The research dataset was mostly published in international journals that had impact factors rated in the 3^rd^ or 4^th^ quartiles, except for one journal (the *cancer* journal, 1^st^ quartile), which may reflect the compromised quality and capacity of research being conducted. Furthermore, four of the top 20 publication outlets were journals from the MENA region i.e., Saudi Arabia, Iraq, and Egypt. These findings align with a previous study showing that the most publications (40%) from the Arab world in the last decade appeared in Q4 journals with only a few publications (0.7%) in the highest quartile score (Q1) journals (26). Increasing investment in research infrastructure and capacity-building initiatives is crucial to improve the quality of scientific material produced in the FCS. Additionally, proper acknowledgement of the contributions of authors from LMICs in the authors’ list is essential, as undervaluing or overlooking their efforts can perpetuate disparities in research recognition and funding.

The majority of publications in cancer research came from institutions in Lebanon, Iraq, and Syria with Lebanon being the most productive country over the analysis period. Surprisingly, our study found no significant correlation between a country’s GDP per capita and its cancer research productivity. Despite having the second-highest GDP per capita, Lebanon exceeded other countries in terms of published cancer research which may be due to active collaboration with foreign researchers and the availability of research infrastructure provided by academic medical centers in Lebanon with strong ties to international academic institutions. These centers, such as the American University of Beirut Medical Center and Hotel Dieu de France, are affiliated with renowned private universities, like the American University of Beirut (AUB) and Université St-Joseph (USJ) (29, 30) that rely on international funding schemes to support their research activities (31, 32). Increasing collaboration and knowledge-sharing across institutions and countries in the region could be an effective strategy to address the gaps in cancer research. The high research productivity in Lebanon may also be due to the country’s physician density, which was 2.1 physicians per 10,000 people in 2018, compared to other countries like Iraq, Libya, Syria, and the West Bank and Gaza (World Bank). It is worth noting that private universities represent over 90% of the higher education institutions in Lebanon (32), but more research is needed to effectively evaluate the impact of private ownership on academic institutions’ scientific productivity.

Our results reveal that there is a significant gender disparity in cancer research in fragile and conflict-affected settings in the MENA region, with around 70% of the first authors being men. This underrepresentation of women in cancer research is consistent with the UNESCO Institute for Statistics (UIS) data from 107 countries, covering the years 2015-2018, in which women made up 33.3% of researchers globally (33). Regionally, in non-conflict settings, Tunisia is the only country where women researchers outnumber men (56% of Tunisian researchers are women), while some countries have made notable strides towards gender equity in research in a short period, including Algeria (from 35% in 2005 to 47% in 2017), Egypt (from 36% in 2007 to 46% in 2018), and Kuwait (from 23% in 2008 to 53% in 2018) (34). Importantly, gender stereotypes and biases, family responsibilities, unequal access to funding, unconscious bias in hiring and promotion, workplace culture, discrimination, and harassment maybe impact women’s representation in research (35), which is further compounded in conflict-affected settings due to political instability, security insurgencies, and weak women-empowering policies (36, 37). Addressing these issues will require policymakers’ attention, and promoting gender equity in cancer research, such as supporting female researchers and addressing biases in the research process, may help to enhance the quality and diversity of research in the region and globally.

The study highlights some gaps and limitations that need to be addressed in future research. The search strategy was restricted to the WoS database and excluded articles written in Arabic which may have led to an underestimation of the total number of published documents. Another limitation is the restriction to the Web of Science database, which excludes other data sources such as Scopus, Google Scholar, Index Medicus, or Microsoft Academic Search, which may have led to an incomplete view of the research output in FCS. Additionally, bibliometric studies concentrate on the research publications’ methodology and designs rather than the importance of their findings. Due to the vast amount and span of articles analyzed, it was not possible to analyze the impact factor and citations of each paper. Finally, due to concurrent conflicts and publication barriers, the study results may not accurately reflect the scope and goal of research in some FCS. Therefore, future research should account for the local context and challenges faced by researchers in conflict-affected regions.

## Conclusion

Despite an increase in cancer research in conflict settings it still falls behind other countries in the MENA region and globally. It is crucial to identify the barriers and challenges to cancer research in conflict settings, expand funding, increased investment in research infrastructure and capacity-building initiatives, and encourage collaborative research to improve both the quantity and quality of research output. This study provides an in-depth understanding of cancer research output in MENA region conflict settings, which can inform the development of evidence-based policies and interventions.

## Data availability statement

The original contributions presented in the study are included in the article/**Supplementary Material**. Further inquiries can be directed to the corresponding author.

## Author contributions

ZS contributed to the conception and design of the study, and data analysis. YY and MH contributed to the data acquisition and analysis, TF and ME did the analysis and interpretation of data. LJ did the graphics. TF, ME, and MK drafted the manuscript. ZS, DM, AT critically revised and oversaw the work. All authors contributed to the article and approved the submitted version.

## Supplementary material

Supplementary Figure 1 Preferred Reporting Items for Systematic Reviews and Meta-Analyses (PRISMA) bibliometric study flowchart.

Supplementary Figure 2 The percentage change in the number of cancer research publications in FCS in the MENA region from 2000 to 2021.

Supplementary Figure 3 The number of publications per 100K people on cancer research in FCS per country and the median GDP per capita (current US$) between 2000 to 2021.

Supplementary Figure 4 Word cloud for top keywords of cancer research in FCS in the MENA region from 2000 to 2021. The importance of these single words is reflected by the font size or color.
