# Supplementary figures and images for: The state of cancer research in fragile and conflict-affected settings in the Middle East and North Africa Region: A bibliometric analysis
Source: Front Oncol. 2023 Mar 23;13:1083836. doi: 10.3389/fonc.2023.1083836 (PMC10076849; doi:10.3389/fonc.2023.1083836)

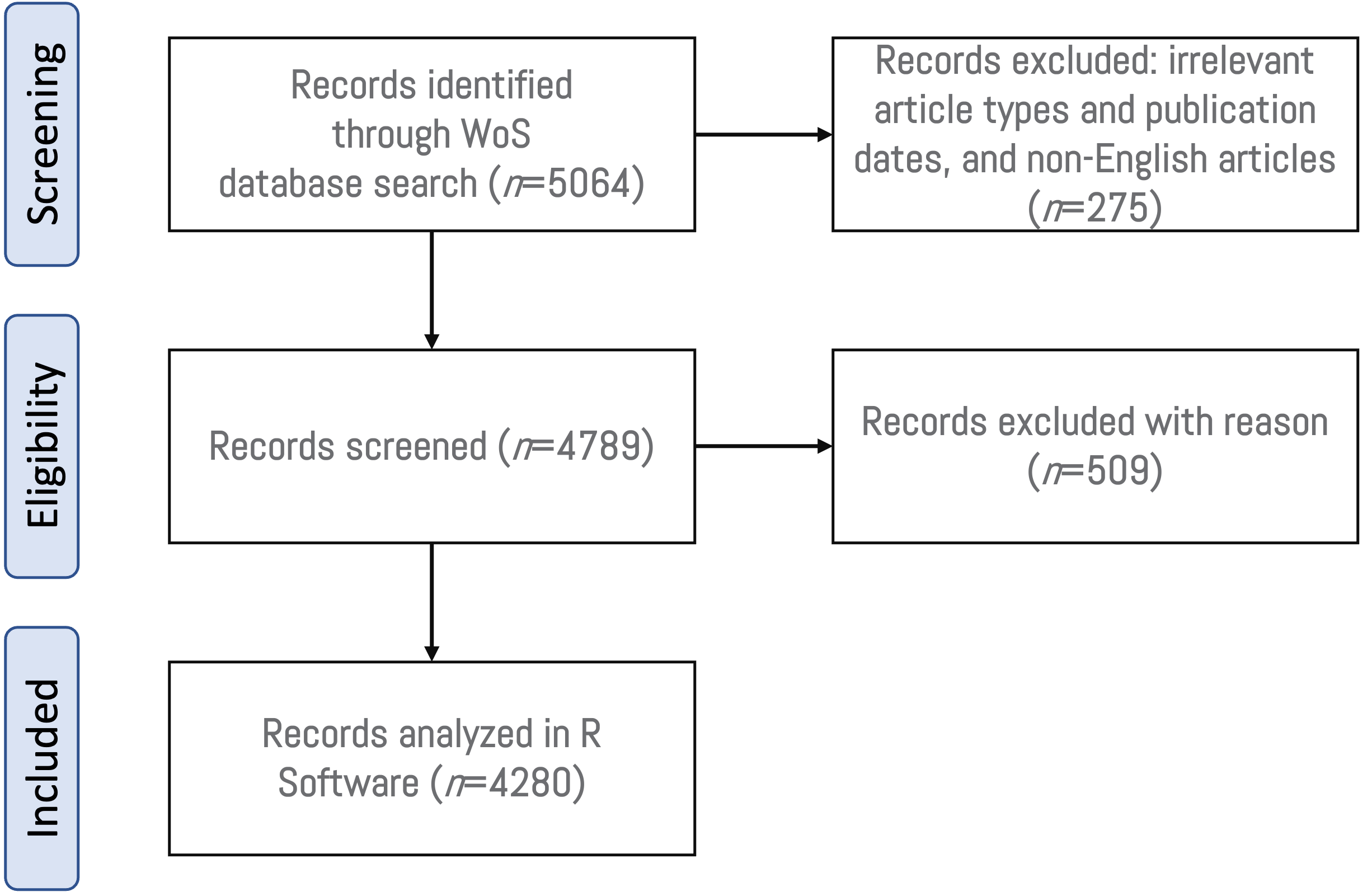

Supplement: Supplementary Figure 1 — Preferred Reporting Items for Systematic Reviews and Meta-Analyses (PRISMA) bibliometric study flowchart. [file Image_1.png]

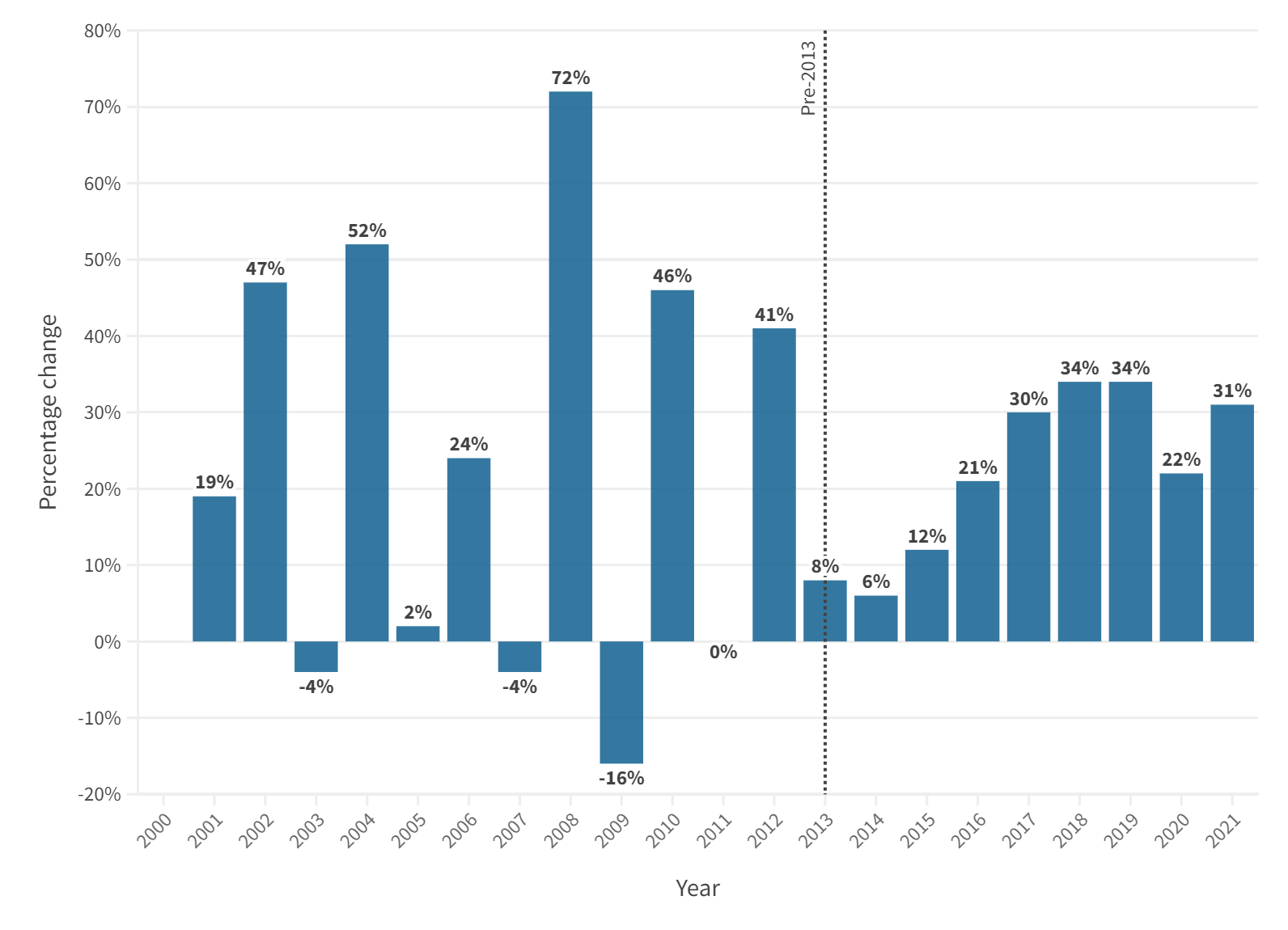

Supplement: Supplementary Figure 2 — The percentage change in the number of cancer research publications in FCS in the MENA region from 2000 to 2021. [file Image_2.jpeg]

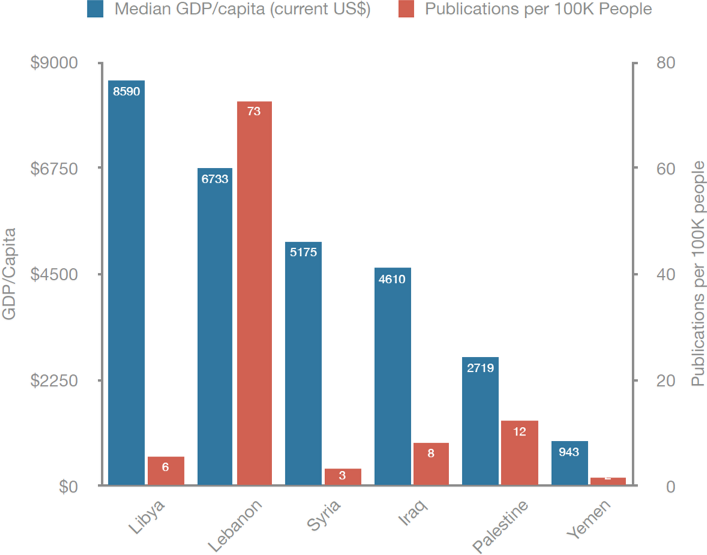

Supplement: Supplementary Figure 3 — The number of publications per 100K people on cancer research in FCS per country and the median GDP per capita (current US$) between 2000 to 2021. [file Image_3.jpeg]

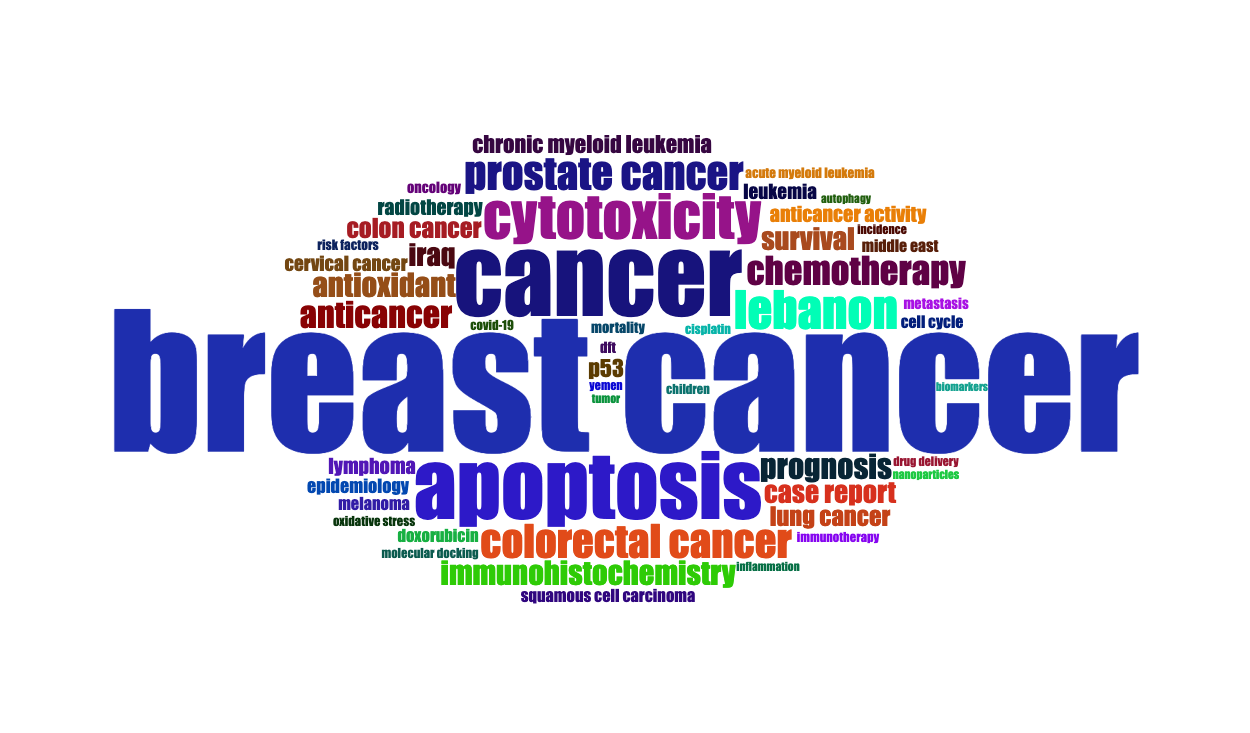

Supplement: Supplementary Figure 4 — Word cloud for top keywords of cancer research in FCS in the MENA region from 2000 to 2021. The importance of these single words is reflected by the font size or color. [file Image_4.jpeg]
