# Supplementary material for: The state of cancer research in fragile and conflict-affected settings in the Middle East and North Africa Region: A bibliometric analysis
Source: Front Oncol. 2023 Mar 23;13:1083836. doi: 10.3389/fonc.2023.1083836 (PMC10076849; doi:10.3389/fonc.2023.1083836)
Supplement: Supplementary file 5 [file DataSheet_1.docx]

**Supplemental Material**

**Supplemental Table 1:** Search Strategy in WoS database

| *Indexes=SCI-EXPANDED, SSCI, A&HCI, ESCI Timespan=2000-2021*  Refined by document types: (ARTICLE OR DATA PAPER OR EARLY ACCESS) |
| --- |
| #1  cu= (Iraq or Lebanon or Libya or Syria or Yemen or Palestine) |
| **AND** |
| #2  (((su=(oncology) or (ts=(*cancer* or neoplas* or *tumo$r* or *carcino* or *metasta* or onco* or chemotherap* or maligna* or *leuk$emi* or lymphom* or *melanoma* or *sarcoma* or hodgkin*or *hemangioma* or *blastoma* or *adenoma* or *prolactinoma* or *nephroma* or *hepatoma* or *luteoma* or *glioma* or *cytoma* or *apudoma* or *gastrinoma* or *ependymoma* or *myeloma* or *chemotherap* or *thymoma* or *meningioma*) ))) or ts=(("multiple pulmonary nodule$") or ("pancoast* syndrome*") or ("solitary pulmonary nodule$") or ("pulmonary coin lesion$") or ("familial polypos*s") or ("gardner syndrome$") or ("lynch syndrome") or ("brill symmers disease") or ("endolymphatic stromal myos*") or ("denys drash syndrome") or ("wilms tumor pseudohermaphroditism") or ("wagr syndrome$") or ("wagr contiguous gene syndrome$") or ("wagr compl*") or ("wilms tumor aniridia genitourinary anomalies mr syndrome") or (insulinoma$) or (insuloma$) or (gastrinoma$) or (glucagonoma$) or (somatostatinoma$) or (vipoma$) or ("pancreatic cholera*") or ("watery diarrhea syndrome") or ("vipoma syndrome") or (wdha) or (wdhh) or ("verner morrison syndrome") or ("watery diarrhea with hypokalemic alkalosis") or ("thyroid nodule$") or ("congenital epuli*") or ("oral leukoplakia$") or ("oral leukokeratos*") or ("keratosis oral") or ("oral hairy leukoplakia$") or (luteinoma$) or ("meig* syndrome") or (thecoma$) or (neurocytoma$) or (pinealoma$) or (pinealocytoma$) or (pineocytoma$) or ("hypothalamic teratoma$") or ("nelson syndrome") or (mesothelioma$) or ("watery diarrhea hypokalemia and achlorhydria syndrome") or ("zollinger-ellison syndrome") or (zes) or (somatostatinoma) or (grfoma) or (acthoma) or (ppoma) or (prolactinoma) or (craniopharyngioma*) or (myelodysplastic) or (myeloproliferative) or (macroglobulin$emi*) or ("mycosis fungoides") or (ependimoma) or ("sezary syndrome") or (craniopharyngioma*) or (myelodysplastic) or (macroglobulin$emi*) )) or ts = ((phyllodes tumo*r$) or (cystosarcoma phyllo$des) or (malignant cystosarcoma phyllodes) or (breast invasive ductal carcinoma) or (infiltrating duct carcinoma$) or (mammary ductal carcinoma$) or (breast cancer) or (breast neoplasm$) or (breast tumo*r$) or (human mammary neoplasm$) or (human mammary carcinoma$) ) or so=(acta-oncologica or advances-in-cancer-biomarkers-frombiochemistry- to-clinic-for-a-critical-revision or advances-in-cancerresearch or advances-in-immunology or american-journal-of-cancerresearch or american-journal-of-clinical-oncology-cancer-clinicaltrials or annals-of-oncology or annals-of-surgical-oncology or anti-cancer-agents-in-medicinal-chemistry or anti-cancer-drugs or anticancer-research or applications-of-viruses-for-cancer-therapy or asian-pacific-journal-of-cancer-prevention or asia-pacific-journal-ofclinical- oncology or biochimica-et-biophysica-acta-reviews-oncancer or biological-basis-of-alcohol-induced-cancer or bloodcancer- journal or bmc-cancer or brain-tumor-pathology or breastcancer or breast-cancer-research or breast-cancer-research-andtreatment or british-journal-of-cancer or bulletin-du-cancer or ca-acancer- journal-for-clinicians or cancer or cancer-and-metastasisreviews or cancer-biology-therapy or cancer-biomarkers or cancerbiotherapy- and-radiopharmaceuticals or cancer-causes-control or cancer-cell or cancer-cell-international or cancer-chemotherapyand- pharmacology or cancer-control or cancer-cytopathology or cancer-discovery or cancer-epidemiology or cancer-epidemiologybiomarkers- prevention or cancer-gene-therapy or cancer-genetics or cancer-genomics-proteomics or cancer-imaging or cancerimmunology- immunotherapy or cancer-immunology-research or cancer-investigation or cancer-journal or cancer-letters or cancermedicine or cancer-nursing or cancer-prevention-research or cancerradiotherapie or cancer-research or cancer-research-and-treatment or cancer-science or cancer-treatment-reviews or carcinogenesis or cell-polarity-and-cancer or cellular-oncology or chemotherapy or chinese-journal-of-cancer or chinese-journal-of-cancer-research or clinical-&-experimental-metastasis or clinical-breast-cancer or clinical-cancer-research or clinical-colorectal-cancer or clinicalgenitourinary- cancer or clinical-journal-of-oncology-nursing or clinical-lung-cancer or clinical-lymphoma-myeloma-leukemia or clinical-oncology or clinical-translational-oncology or criticalreviews- in-oncology-hematology or current-advances-inosteosarcoma or current-cancer-drug-targets or currentoncology or current-oncology-reports or current-opinion-inoncology or current-problems-in-cancer or current-treatmentoptions- in-oncology or endocrine-related-cancer or europeanjournal- of-cancer or european-journal-of-cancer-care or europeanjournal- of-cancer-prevention or european-journal-ofgynaecological- oncology or european-journal-of-oncology or european-journal-of-oncology-nursing or expert-review-ofanticancer- therapy or familial-cancer or future-oncology or gastric-cancer or genes-chromosomes-cancer or guidancemolecules- in-cancer-and-tumor-angiogenesis or gynecologiconcology or head-neck-oncology or hematological-oncology or hematology-oncology-clinics-of-north-america or hereditarycancer- in-clinical-practice or hormones-cancer or immunity-tolisteria- monocytogenes or indian-journal-of-cancer or infectiousagents- and-cancer or inflammation-and-cancer or integrativecancer- therapies or international-journal-of-cancer or international-journal-of-clinical-oncology or internationaljournal- of-gynecological-cancer or international-journal-ofoncology or international-journal-of-radiation-oncologybiology- physics or japanese-journal-of-clinical-oncology or jncijournal- of-the-national-cancer-institute or journal-of-adolescentand- young-adult-oncology or journal-of-bone-oncology or journal-of-breast-cancer or journal-of-cancer or journal-ofcancer- education or journal-of-cancer-research-and-clinicaloncology or journal-of-cancer-research-and-therapeutics or journal-of-cancer-survivorship* or journal-of-chemotherapy or journal-of-clinical-oncology or journal-of-environmentalpathology- toxicology-and-oncology or journal-of-experimentalclinical- cancer-research or journal-of-geriatric-oncology or journal-of-gynecologic-oncology or journal-of-hematologyoncology or journal-of-medical-imaging-and-radiation-oncology or journal-of-neuro-oncology or journal-of-pediatric-hematologyoncology or journal-of-pediatric-oncology-nursing or journal-ofpsychosocial- oncology or journal-of-surgical-oncology or journal-of-the-national-cancer-institute or journal-of-thenational- comprehensive-cancer-network or journal-of-thoraciconcology or lancet-oncology or leukemia or leukemia-lymphoma or leukemia-research or lung-cancer or medical-oncology or melanomaresearch or microrna-cancer-from-molecular-biology-to-clinicalpractice or molecular-cancer or molecular-cancer-research or molecular-cancer-therapeutics or molecular-carcinogenesis or molecular-oncology or nature-reviews-cancer or nature-reviewsclinical- oncology or neoplasia or neoplasma or neuroendocrinetumors- a-multidisciplinary-approach or neuro-oncology or nutrition-and-cancer-an-international-journal or oncogene or oncogenesis or oncoimmunology or oncologie or oncologist or oncology or oncology-letters or oncology-new-york or oncology-nursing-forum or oncology-reports or oncologyresearch or oncology-research-and-treatment or oncotarget or oncotargets-and-therapy or onkologe or onkologie or oraloncology or pathology-oncology-research or pediatric-bloodcancer or pediatric-hematology-and-oncology or pigment-cellmelanoma- research or progress-in-tumor-research or prostatecancer- and-prostatic-diseases or psycho-oncologie or psychooncology or radiation-oncology or radiology-and-oncology or radiotherapy-and-oncology or recent-patents-on-anti-cancerdrug- discovery or renaissance-of-cancer-immunotherapy or seminarsin- cancer-biology or seminars-in-oncology or seminars-in-radiationoncology or strahlentherapie-und-onkologie or successes-andlimitations- of-targeted-cancer-therapy or supportive-care-in-cancer or surgical-oncology-clinics-of-north-america or surgicaloncology- oxford or targeted-oncology or technology-in-cancerresearch- treatment or therapeutic-advances-in-medical-oncology or thoracic-cancer or translational-oncology or tumor-biology or tumori or tumor-microenvironment-and-cellular-stress-signalingmetabolism- imaging-and-therapeutic-targets or uhod-uluslararasihematoloji- onkoloji-dergisi or urologic-oncology-seminars-andoriginal- investigations or veterinary-and-comparative-oncology or world-journal-of-surgical-oncology or wspolczesna-onkologiacontemporary- oncology) or ti=((antitumor* not necrosis) or (hpv* not parvo*) or (irradiation and fractionated) or (myc not (c or n) ) or (pml and (apopto* or gene or nuclear or nucleus or protein* or rar* or ubiquitin)) or (topoisomerase and inhibitor) or (tumo*r* not necrosis) or 5t4 or adenocarcinoma or adenocarcinomas or adenoma or adenomas or adenosarcomas or adenosarcoma or adriamycin or agr3 or akap13 or alemtuzumab or alex2 or alitretinoin or altretamine or ameloblastoma or ameloblastomas or amifostine or aml or anastrozole or angiosarcoma or angiosarcomas or anticancer* or anticarcino* or antileukemic or antimelanoma or antimyeloma or antineoplas* or antiprolif* or antitumo*r or arimidex* or armcx1 or aromatase or astrocytoma or astrocytomas or azacitidine or b-8801 or bcar1 or bcl2 or bcl2 or bcr-abl or bcr/abl or bicalutamide or bin2 or bioreductive or bleomycin or bortezomib or braf or brap1 or brca or brca1 or brca2 or brcc3 or brachytherapy or bri3bp or brms1 or bryostatin* or busulfan or c2orf40 or caelyx* or cage1 or cage-1 or cancer* or capecitabine or carbogen or carboplatin or carcino* or carmustine or cdkn2a or cdx2 or cea or cep290 or cervical smear or cetuximab or chemoprevent* or chemoradiotherapy or chemosensitiv* or chemotherap* or chlorambucil or cholangiocarcinoma or cholangiocarcinomas or chondrosarcoma or chondrosarcomas or choriocarcinoma or choriocarcinomas or cin or cisplatin or cladribine or cll or cml or combretastatin or craniopharyngioma or craniophar yngiomas or ct45-1 or ct47 or cyclophosphamide or cystadenocarcinoma or cystadenocarcinomas or cystadenomas or cystadenoma or cytarabine or cytosine-arabinoside or dacarbazine or dasatinib or daunorubicin or dbc1 or ddx53 or decitabine or dermatofibrosarcoma or dermatofibrosarcomas or docetaxel or doxorubicin* or du-pan-2 or dysgerminoma or dysgerminomas or ebag9 or ecrg or eef1a1 or elac2 or eortc or ependymoma or ependymomas or epirubicin or erbb* or erlotinib or estramustine or etoposide* or etv2 or exemestane or fibroma or fibromas or fibrosarcoma or fibrosarcomas or fli1 or floxuridine or fluorouracil or fos or fulvestrant or ga50 or ganglioglioma or gangliogliomas or ganglioneuroblastoma or ganglioneuroblastomas or gefitinib or gemcitabine or gemtuzumab or germinoma or germinomas or gleason or glevec* or glioblastoma or glioblastomas or glioma or gliomas or gliosarcoma or gliosarcomas or glivec or goserelin or hccr1 or hemangioblastoma hemangioblastomas or hemangioendothelioma or hemangioendotheliomas or hemangiosarcoma or hamangiosarcomas or hepatoblastoma or hepatoblastomas or hepatocarcino* or hepatoma or hepatomas or her2 or herceptin* or histiocytoma or histiocytomas or hodgkin disease or hodgkins or hrpt2 or hydroxyurea or hypernephroma or hypernephromas or ibritumomab-tiuxetan or idarubicin or ifos*amide* or imatinib or imrt or insulinoma or insulinomas or intratumor* or iodine-131-anti-b1- antibody or ipilimumab or iressa or irinotecan or ixabepilone or jun or l514s or l552s or lapatinib or lcap or leiomyoma or leiomyomas or leiomyosarcoma or leiomyosarcomas or lenalidomide or letmd1 or letrozole or leukaem* or leukem* or li fraumeni or liposarcoma or liposarcomas or lomustine or ly2k or lymphoblastic or lymphoma* or lymphoproliferative or macc1 or malignanc* or malignant or mammogra* or map3k8 or mastectom* or medulloblastoma or medulloblastomas or melanoma or melanomas or melphalan* or meningioma or meningiomas or mercaptopurine or mesothelioma or mesotheliomas or metastas* or metastat* or methylguanine or mitomycin or mitoxantrone or mlh1 or mll or msh or msh2 or muc1 or myb or myelodysplas* or myeloid or myeloma* or myeloproliferative or myxofribosarcoma or myxofribosarcomas or neoplas* or nephroblastoma or nephroblastomas or nephroma or nephromas or neurinoma or neurinomas or neuroblastoma or neuroblastomas or neurofibrosarcoma or neurofibrosarcomas or neuroma or neuromas or nhl or nsclc or nup98 or oligoastrocytoma or oligoastrocytomas or oligodendroglioma or oligodendrogliomas or oncogen* or oncolog* or oncolytic or oncoprotein or osteosarcoma or osteosarcomas or oxaliplatin or p12ink4a or panitumab or pap-smear or papilloma or papillomas or pax3 or pbov1 or pegaspargase or pemetrexed or pentostatin or peutz or pheochromocytoma or pheochromocytomas or phtotodynamictherap* or plasmacytoma or plasmacytomas or pml/rar* or pms1 or polycythemia-rubra or pphln1 or prednisolone or procarbazine or prostatectomy or protooncogen* or prostate-specific-antigen or pten or rad51 or radiation-therapy or radiosensi* or radiosurgery or radiotherap* or raltitrexed or ras or rb1 or rcvrn or ret or retinoblastoma* or rhabdomyosarcoma or rhabdomyosarcomas or rhobtb2 or sarcoma or sarcomas or schwannoma or schwannomas or sdccag or seminoma or seminomas or sfxn4 or skcg-1 or slc35c2 or sncg or sorafenib or spanxc or src or steap2 or streptozocin or sunitinib or tamoxifen or tarceva or taxol or taxotere or tbc1d3 or tccsg or temodal or temozol*mide or temsirolimus or teniposide or teratoma or teratomas or tff1 or thioguanine or thiotepa or thymoma or thymomas or tomotherapy or tomudex or topotecan or tp53 or trastuzumab or treosulfan or trovax or tsc1 or uoeh-lc-1 or vcrp protocol or vinblastine or vincristine or vinorelbine or vwa5a or waldenstrom* or xage1a or xeroderma-pigmentosum or zoledronicacid or abiraterone or anthracycline or anthracyclines or antileukemia or axitinib or blinatumomab or bosutinib or brentuximab or carfilzomib or catumaxomab or cediranib or ceritinib or chemoradiation or chordoma or chordomas or crizotinib or cystectomy or dcis or dinaciclib or dovitinib or enzalutamide or eribulin or esophagectomy or esthesioneuroblastoma or esthesioneuroblastomas or fungoides or (gist and gastr*) or hcc or hnscc or ibrutinib or idelalisib or lipoblastoma or lipoblastomas or lymphadenectomy or lynch-syndrome or nilotinib or oesophagectomy or osteochondroma or osteochondromas or paclitaxel or pancreaticoduodenectomy or pancreatoblastoma or pancreatoblastomas or pancreatoduodenectomy or panitumumab or paraneoplastic or pazopanib or postmastectomy or proton-beamtherapy or pseudomyxoma or pseudomyxomas or regorafenib or sbrt or trametinib or vemurafenib or vismodegib or vmat) |

**Supplemental Table 2:** Summary of the Main information of collected bibliometric data in FCS in the MENA region from 2000 to 2021.

| Documents | 4,280 |
| --- | --- |
| Sources (Journals, Books, etc.) | 1,514 |
| Keywords Plus (ID) | 8,896 |
| Author's Keywords (DE) | 9,560 |
| Period | 2000-2021 |
| Average citations per document | 18.1 |
| Average citations per year per document | 2.718 |
| Authors | 21,587 |
| Authors of single-authored documents | 100 |
| Authors of multi-authored documents | 29,146 |
| Authors per document | 6.83 |
| Collaboration Index | 7.01 |

**Supplemental Table 3.** Top 20 most relevant sources by the number of documents, Impact Factor (IF), and quartile (Q) ranking published on cancer research in FCS in the MENA region from 2000 to 2021.

| **Sources** | **Articles** | **IF (2021)** | **Q (2021)** |
| --- | --- | --- | --- |
| International Journal of Surgery Case Reports | 53 | 0.690 | Q3 |
| PLOS One | 48 | 3.752 | Q2 |
| Saudi Medical Journal | 45 | 1.422 | Q4 |
| Scientific Reports | 44 | 4.996 | Q2 |
| Iraqi Journal of Hematology | 37 | N/A | N/A |
| Pakistan Journal of Medical and Health Sciences | 35 | 0.073 | Q3 |
| Molecules | 34 | 4.927 | Q2 |
| Annals of Medicine and Surgery | 32 | 1.346 | Q2 |
| Bone Marrow Transplantation | 27 | 5.176 | Q2 |
| BMC Cancer | 26 | 4.638 | Q2 |
| Cancer | 26 | 6.921 | Q1 |
| Oncology Letters | 26 | 3.111 | Q3 |
| Asian Pacific Journal of Cancer Prevention | 25 | 2.514 | Q3 |
| Baghdad Science Journal | 25 | 0.728 | Q4 |
| Cureus | 25 | 1.150 | Q3 |
| Egyptian Journal of Chemistry | 24 | 1.539 | Q3 |
| Frontiers in Oncology | 24 | 5.738 | Q2 |
| Research Journal of Pharmaceutical Biological and Chemical Sciences | 24 | 0.942 | Q4 |
| Biomed Research International | 23 | 3.246 | Q3 |
| Pediatric Blood & Cancer | 23 | 3.838 | Q2 |
